# In vivo Quantification of the Effects of Radiation and Presence of Hair Follicle Pores on the Proliferation of Fibroblasts in an Acellular Human Dermis in a Dorsal Skinfold Chamber: Relevance for Tissue Reconstruction following Neoadjuvant Therapy

**DOI:** 10.1371/journal.pone.0125689

**Published:** 2015-05-08

**Authors:** Mario Vitacolonna, Djeda Belharazem, Patrick Maier, Peter Hohenberger, Eric Dominic Roessner

**Affiliations:** 1 Division of Surgical Oncology and Thoracic Surgery, Department of Surgery, University Medical Centre Mannheim, University of Heidelberg, Germany; 2 Institute of Pathology, University Medical Centre Mannheim, University of Heidelberg, Germany; 3 Department of Radiation Oncology, University Medical Center Mannheim, University of Heidelberg, Germany; National Institutes of Health, UNITED STATES

## Abstract

**Introduction:**

In neoadjuvant therapy, irradiation has a deleterious effect on neoangiogenesis. The aim of this study was to examine the post-implantation effects of neoadjuvant irradiation on the survival and proliferation of autologous cells seeded onto an acellular human dermis (hAD; Epiflex). Additionally, we examined the influence of dermal hair follicle pores on viability and proliferation. We used dorsal skinfold chambers implanted in rats and *in-situ* microscopy to quantify cell numbers over 9 days.

**Methods:**

24 rats received a skinfold chamber and were divided into 2 main groups; irradiated and unirradiated. In the irradiated groups 20Gy were applied epicutaneously at the dorsum. Epiflex pieces were cut to size 5x5mm such that each piece had either one or more visible hair follicle pores, or no such visible pores. Fibroblasts were transduced lentiviral with a fluorescent protein for cell tracking. Matrices were seeded statically with 2.5x10^4^ fluorescent fibroblasts and implanted into the chambers. In each of the two main groups, half of the rats received Epiflex with hair follicle pores and half received Epiflex without pores. Scaffolds were examined *in-situ* at 0, 3, 6 and 9 days after transplantation. Visible cells on the surface were quantified using ImageJ.

**Results:**

In all groups cell numbers were decreased on day 3. A treatment-dependent increase in cell numbers was observed at subsequent time points. Irradiation had an adverse effect on cell survival and proliferation. The number of cells detected in both irradiated and non-irradiated subjects was increased in those subjects that received transplants with hair follicle pores.

**Discussion:**

This *in-vivo* study confirms that radiation negatively affects the survival and proliferation of fibroblasts seeded onto a human dermis transplant. The presence of hair follicle pores in the dermis transplants is shown to have a positive effect on cell survival and proliferation even in irradiated subjects.

## Introduction

Decellularized scaffolds derived from biological tissue such as acellular dermis are being increasingly used in regenerative medicine research [1–3], Reports of pre-clinical studies and clinical applications are becoming widespread [4–9]. Studies have shown that the implantation of cell-augmented matrices can enhance wound healing [10–15].

Decellularization processes should leave an intact extracellular matrix (ECM) structure and retain the desirable mechanical properties of the native tissue. Functional proteins, such as collagen, fibronectin, hyaluronic acid, laminin, vitronectin, osteopondin and the basement membrane can be preserved [16, 17]. However, the retention of a dense native tissue structure such as is found in the dermis, can limit the extent to which the matrix can be repopulated by cells [16, 18].

Furthermore, the absence of a vascular network within the transplant may have a negative effect on the proliferation and survival of the autologous cells in the graft due to an inadequate supply of cells with nutrients and gas exchange [19, 20].

In addition, the effects of irradiation as a part of a neoadjuvant therapy on cell proliferation in the graft still remain to be explained.

In order to examine the in-vivo survival and proliferation of autologous cells seeded onto an acellular human dermis after irradiation, a dorsal skin-fold chamber model was established in rats. The proliferation of autologous cells transfected with a fluorescent plasmid was investigated in-vivo using intravital microscopy. The influence of the presence of hair follicle pores on the acellular dermis on cell survival was also investigated.

## Materials and Methods

### Animals

Thirty-six male Fisher-344 rats (150g, Charles-River, Germany) were used in this study. All animals were held in the vivarium of the University Medical Centre Mannheim. The study was approved by the state authorities (Regierungspraesidium Karlsruhe, Germany: AZ 35–9185.81/G-187/09).

### Human acellular dermis

Human acellular dermis (hAD) (Epiflex, German Institute for Cell and Tissue Replacement, Berlin, Germany) was used. The mechanical processing, decellularization, sterilization, preservation [21], composition [17] and biomechanical properties [22] are described in detail elsewhere.

### Study design

The study comprised 4 treatment groups. The subjects were initially divided into 2 groups of equal size (12 subjects each). In one of these two main groups, all subjects received radiation prior to implantation of the skin-fold chamber. This treatment was selected to serve as an analogue of a neoadjuvant oncological therapy. All subjects received a hAD transplant seeded with fibroblasts transfected with the tomato plasmid. In each of the two main groups, half of the subjects received a transplant containing hair follicle pores and half received a transplant lacking hair follicle pores. The subjects were investigated on day 0, 3, 6 and 9 after transplantation. The subjects were sacrificed thereafter. Table 1 details the study design.

**Table 1:** Tabular depiction of the experimental design. Each group consists of n = 6 animals.

Group	Pores	Radiation	Abbreviation
**1**	+	-	+por-rad
**2**	-	-	-por-rad
**3**	+	+	+por+rad
**4**	-	+	-por+rad

### Cell isolation and culture conditions

Autologous rat fibroblasts were obtained from subcutaneous fat as described previously [23]. Briefly, the adipose tissue was digested using 2mg/ml collagenase type 2 (PAA, Germany) at 37°C for 2h with vigorous shaking to obtain a single cell suspension. The suspension was washed twice with Dulbecco’s modified Eagle medium (DMEM) with 4,5g/L glucose (PAA, Germany) containing 10% (v/v) fetal bovine serum (FBS) (PAA, Germany). After digestion the suspension was centrifuged at 400g for 5 min. The resultant cell pellet was plated onto 100 mm^2^ tissue culture plates (Greiner Bio One, Germany) supplemented with DMEM (with 10% FBS and 1% penicillin/strepavidin solution (PAA, Germany)) and maintained at 37°C in an incubator with 5% CO_2_. The medium was changed every 3 days, passages were carried out at 70% to 80% confluence.

### Vector plasmids and viral vector production

The lentiviral plasmid pHR’SINcPPT-SEW, the envelope plasmid pMD.G, and the multi-deleted packaging plasmid pCMVR8.91 have been described previously [24, 25]. The plasmid containing the coding sequence of tdTomato has also been described elsewhere [26]. The tdTomato sequence was cloned via BamHI/EcoRI into the expression vector pcDNA3.1. Thus, tdTomato could by excised with BamHI and EcoRI and ligated into pcDNA3.1 vector (LifeTechnologies, Germany) cut with the same restriction enzymes. Subsequently, the tdTomato was cloned via BamHI/XbaI into a modified version of the lentiviral vector pHR’SINcPPT-SEW (with a deleted XbaI-site 3’ of the 3’LTR) generating pHR’SIN-tomato [27]. A lentiviral supernatant of pHR’SIN-tomato was produced and titrated as described before [28].

### Lentiviral transduction of the fibroblasts

Transduction of the fibroblasts cells was performed as described previously [27]. Briefly, fibroblasts were plated at a density of 1x10^5^ cells/well in a 6-well plate in DMEM. The next day cells were transduced once with lentiviral vector particles with a MOI of 10 in the presence of 8 μg/ml polybrene. After 24 h, the medium was changed to polybrene-free DMEM and after a further overnight incubation the cells were trypsinized, seeded into a T25-flask, and cultured for 5 days. The cells were then sorted with flow cytometry (FlowCore, Center for Biomedicine and Medical Technology, University Medical Centre Mannheim, FACSAria-I cell sorter (Becton-Dickinson, USA) to exclude the non-transduced cells.

### Irradiation

Twelve animals were assigned to the irradiation group. The dorsum of the anesthetized animals was shaved and the dorsal skin was positioned under the irradiation apparatus using two 3/0 Vicryl sutures (Ethicon, USA) and surgical clips. 20 gray were administered epicutaneously 14 days prior to the implantation of the chamber in a single dose with an Intrabeam device (PEC Photoelectronic Corporation PRS400; Voltage 50kV, Current 40μA; Run Time 6min).

### Implantation of the dorsal skin-fold chamber

The surgical implantation of the skinfold chamber has been described previously [29]. Briefly, the rats were anaesthetized via an intraperitoneal injection of 2% Xylazin (Rompun; Bayer, Germany) 5mg/kg and Ketamin (Ketanest; Parke Davis, Germany) 100mg/kg body weight. The animals were placed on a pre-warmed operating table, the back skin was shaved, depilated and disinfected. The dorsal skinfold was stretched using two 3/0 Vicryl sutures (Ethicon, USA). Subsequently, the titanium base plate was sutured to the stretched skin. Using an operation microscope (Zeiss OPMI 9-FC, Zeiss, Germany) an area (15 mm diameter) of the top layer of the dorsal skinfold (skin and subcutaneous tissue with the cutaneous trunci muscle) was circularly excised and the underlying fascial layers were removed carefully from the thin skin muscle layer (panniculus carnosus). The remaining layer of the striated muscle, the subcutaneous tissue, the dermis and the epidermis, were covered with the counterpart of the dorsal skinfold chamber, screwed together and stitched with a 5/0 vicryl suture (Ethicon, USA). Thereafter the observation window was filled with Ringer's solution and covered with a detachable cover glass (Menzel, Germany) avoiding air bubble formation. After preparation, the animals were allowed to recover from anaesthesia and surgery for 48h before the implantation of the scaffolds.

During the period of study, the chambers and the implanted biomaterial underwent qualitative daily assessment. Animals with pathological findings or chamber defects were excluded from the experiment. Inflammation, haemorrhage, oedema and persisting restriction of movement with consequent impairment of nourishment were termination criteria.

### Fibroblast seeding on acellular dermis

Transduced fibroblasts from passage 5 were used for seeding. Epiflex transplants with a thickness of <0.3mm were cut into 5mm x 5mm hAD pieces and allowed to rehydrate in Ringer´s solution for 2h at 37°C in 48 well plates (Greiner, Germany). Pore-containing hAD pieces were cut from the transplants such that each piece contained three pores with a similar diameter in each region of interest (ROI). The rehydrated hAD were initially degassed with a chamber evacuation method to remove air trapped within the matrix [30, 31] and subsequently statically seeded with 300ul of a cell suspension containing 2.5x10^4^ viable fibroblasts. Culture plates were incubated after the seeding procedure at 37°C with 5% CO_2_ for 2h to enable cell attachment. Immediately thereafter, the matrices were implanted into the skinfold chamber.

### Implantation of the seeded dermis

In order to implant the matrices, the animals were anesthetized as described before. The cover slip was removed, the implant was attached to the striated muscle in the centre of each chamber window with an 8/0 suture (Ethicon, USA) and the chamber was sealed and the window was closed with a sterile coverslip, once again avoiding air bubbles.

### In-situ fluorescence microscopy

Microscope examination was conducted under anesthesia (as described above) on days 0, 3, 6 and 9 using an Axiotech Vario 100 intravital microscope (Zeiss, Germany). The 4 borders of the implanted dermis were defined as regions of interest (ROIs). Five pictures were taken from each ROI using a 5x objective (each ROI corresponded an area of 5mm x 1mm). The images were captured with a digital camera (AxioCam ICm1, Zeiss, Germany). To create overlay images of the dermis and the transduced cells, a two-step excitation process was used, the intrinsic fluorescence of the matrix was observed under light filtered at 488nm, then the fluorescence of the fibroblasts was observed under light filtered at 540nm. Images were acquired with Axiovision LE V4.8.2 software (Zeiss, Germany). After overlaying the images with Paint.Net (V3.5, Microsoft, USA), the overlays were stitched together using ICE (Image Composite Editor, V1.4.4.0, Microsoft, USA) in order to create an edge-to-edge view of the ROI.

### Quantification of cell proliferation

To quantify the visible cells within each ROI, the cells were counted manually using ImageJ (National Institute of Health, USA). A total of n = 24 ROIs per group were evaluated.

### Statistics

All statistical tests were conducted with GraphPad Prism V6 (GraphPad Software Inc, USA). A double-sided student’s t-test (confidence level 95%) was used for comparisons of 2 groups.

## Results

### Influence of irradiation on the proliferation of seeded fibroblasts

**Fig 1:**
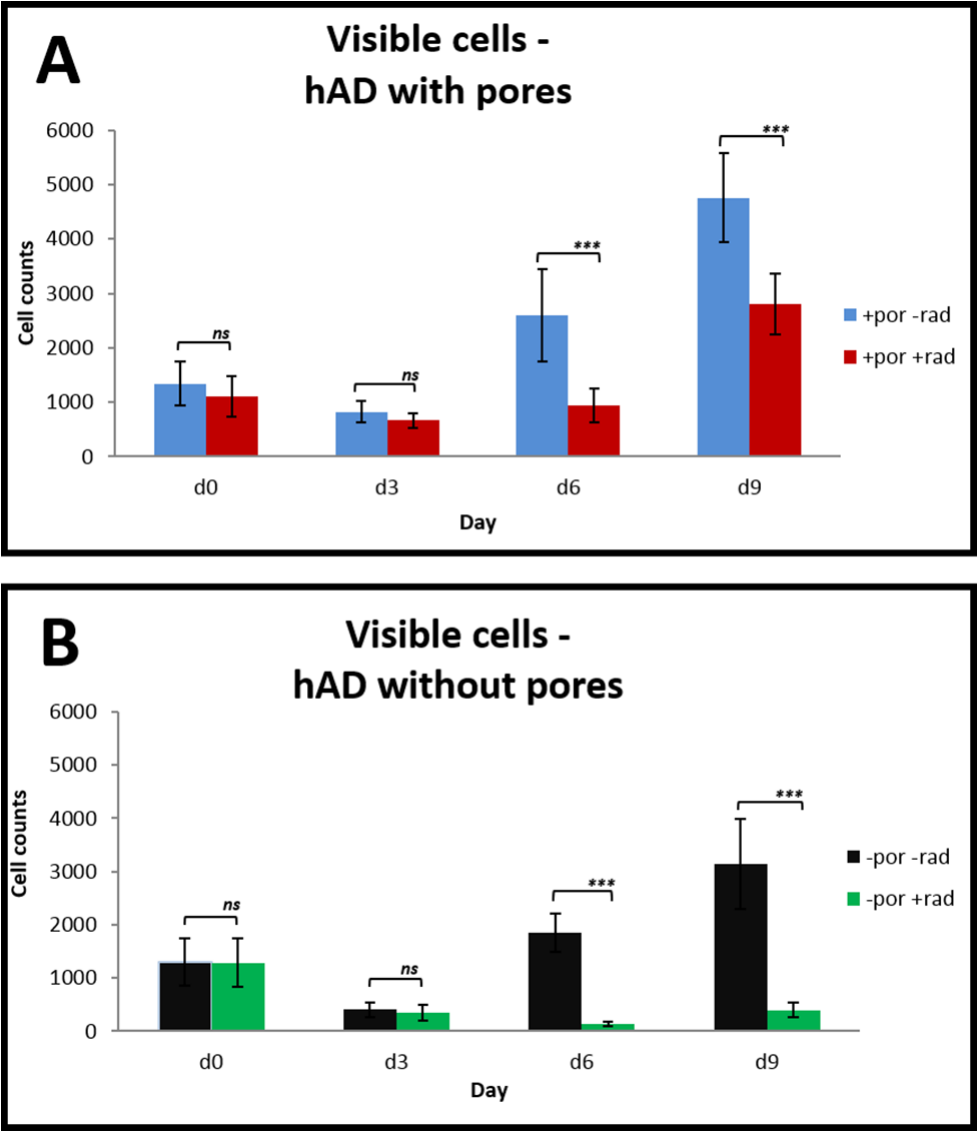
Influence of radiation on cell proliferation. Comparison between (**A**) porous group 1 (+por-rad) and group 3 (+por+rad) and (**B**) non-porous group 2 (-por-rad) and group 4 (-por+rad) respectively with and without radiation. All values are expressed as mean values ± standard deviation.

Fig 1A displays the recorded cell counts on hAD pieces with hair follicle pores from groups 1 (no irrdadiation) and 3 (irradiated).

At day 0 there was no significant difference between the two groups (group 1:1335 ± 403 cells/ROI and group 3: 1103 ± 375 cells/ROI). At day 3 cell numbers decreased similarly in both groups (group 1: 820 ± 199 cells/ROI and group 3: 665 ± 137 cells/ROI). At day 6 a significant difference was observed (group 1: 2592 ± 845 cells/ROI and group 3: 946 ± 310 cells/ROI (p <0.0001)). At day 9 4751 ± 817 cells/ROI could be counted in group 1, whereas 2795 ± 559 cells/ROI were found in group 3 (p <0.0001).

Fig 1B shows the cell counts from hAD pieces without hair follicle pores from groups 2 (no irradiation) and 4 (irradiated).

At day 0 and day 3 there were no significant differences between groups 2 and 4 (Day 0: group 2; 1294 ± 449 cells/ROI and group 4; 1286 ± 449 cells/ROI. Day 3: group 2; 403 ± 136 cells/ROI and group 4: 342 ± 153 cells/ROI). At day 6 there significant difference between the two groups (group 2: 1847 ± 358 cells/ROI and group 4: 130 ± 45 cells/ROI (p <0.0001)). At day 9 there were 3142 ± 842 cells/ROI in group 2 and 397 ± 139 cells/ROI in group 4 (p <0.0001).

### Influence of the presence of hair follicle pores on the proliferation of seeded fibroblasts

**Fig 2:**
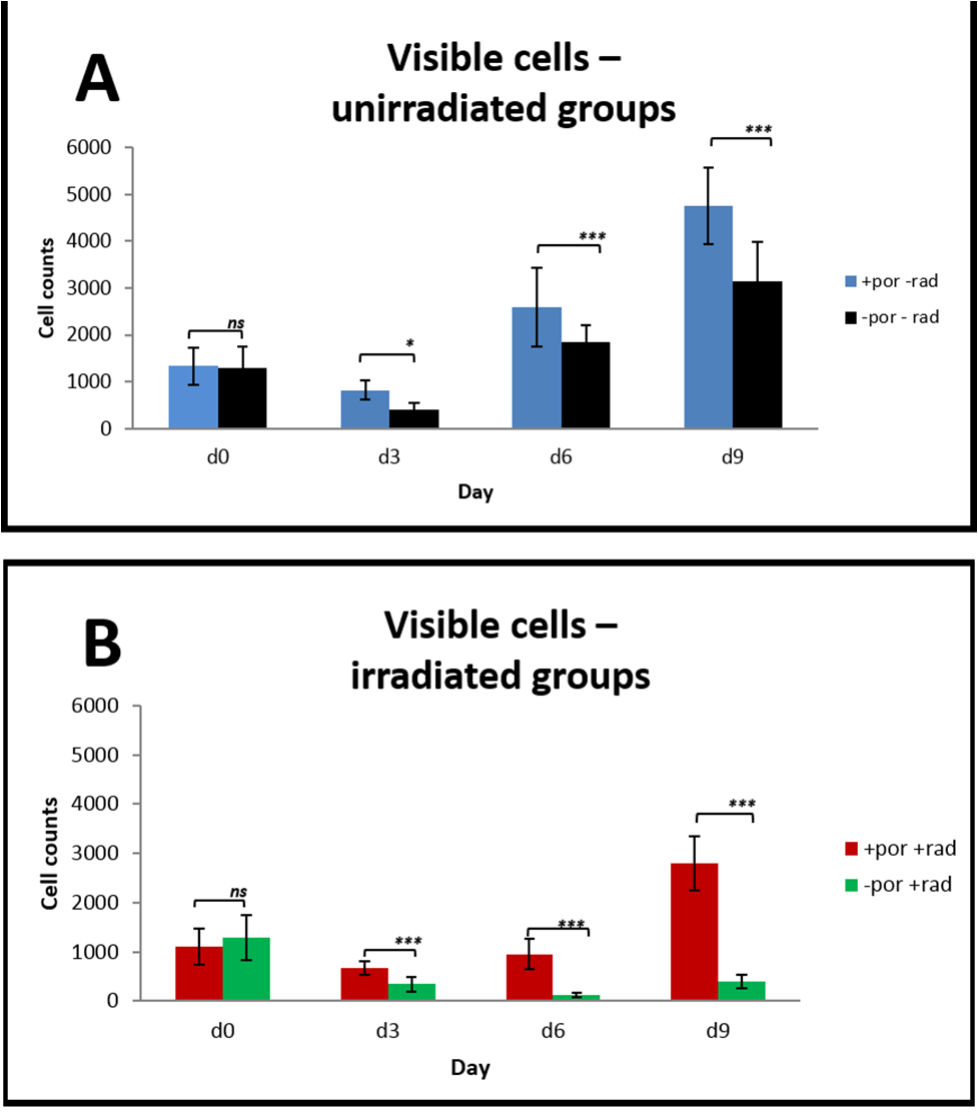
Influence of pores on cell proliferation. Comparison between (**A**) unirradiated group 1 (+por-rad) and group 2 (-por-rad) and (**B**) irradiated group 3 (+por+rad) and group 4 (-por+rad) respectively with and without pores. All values are expressed as mean values ± standard deviation.

Fig 2A depicts the cell counts from the non-irradiated groups 1 (with hair follicle pores) and 2 (without hair follicle pores).

At days 3 (p = 0.0108), 6 (p<0.0001) and 9 (p<0.0001) there were significantly more cells in group 1 (820 ± 199 cells/ROI, 2592 ± 845 cells/ROI and 4751 ± 817 cells/ROI respectively) than in group 2 (403 ± 136 cells/ROI, 1847 ± 358 cells/ROI and 3142 ± 842 cells/ROI).

Fig 2B provides the results of cell quantification in the irradiated groups 3 (with hiar follicle pores) and 4 (without hair follicle pores). Starting at day 3 a significant difference between the two groups was observed (group 3: 665 ± 137 cells/ROI and group 4: 342 ± 153 cells/ROI (p <0.0001)). At day six 946 ± 310 cells/ROI were counted in group 3 and 130 ± 45 cells/ROI in group 4 (p <0.0001). At day 9 in group 3 there were 2795 ± 559 cells/ROI) whereas in group 4) there were 397 ± 139 cells/ROI (p <0.0001).

**Fig 3:**
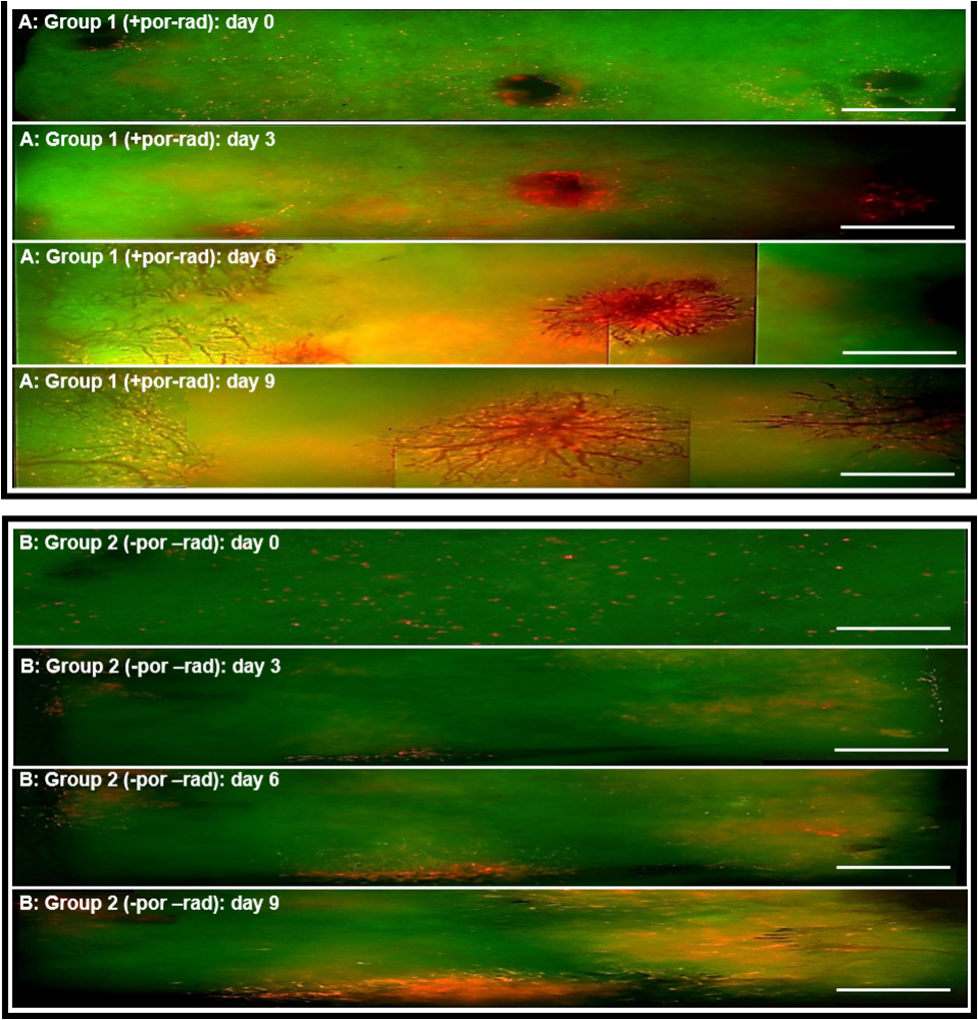
Representative intravital microscopic images. Intravital microscopic overlay images of (A) group *1 (+pores/-rad) and (B) group 2 (-pores/-rad) at day 0–9*. The single images were taken with a 5x objective and stitched together with Microsoft Image Composite Editor (ICE) in order to obtain an edge-to-edge view of the ROI. The green background represents the intrinsic fluorescence of the dermis and the transduced fibroblasts are light red.

Fig 3 provides sample microscopy images from the non-irradiated groups 1 (with hair follicle pores) and 2 (without pores) at days 0 to 9.

After cell seeding (day 0) the cells were reasonably evenly distributed across the hAD pieces in both groups.

In group 1 at day 3 cell density appears to be decreased at points far from borders and hair follicle pores, but appears to be increased in the vicinity of pores and at the edge of the hAD pieces. There also appears to be greater proliferation of cells in the vicinity of hair follicle pores and at the edges of the hAD pieces than in zones far from pores and borders on days 6 and 9 (Fig 3A).

At day 3 the majority of the cells remaining in group 2 were located at the border. In the center of the hAD pieces cell numbers appear to be diminished. At days 6 and 9 there appears to be more cell proliferation close the borders than in the central areas.

## Discussion

An investigation of the post-implantation proliferation of fibroblasts seeded onto human acellular dermis is described. The effect of pre-implantation irradiation (intended to model a multi-modal neoadjuvant therapeutic approach) and the effect of the presence of hair follicle pores on fibroblast survival and proliferation were assessed.

Previous studies investigated cell proliferation in decellularized dermis and demonstrated that cells including autologous fibroblasts can survive and proliferate in such transplants [32–37]. This paper describes the first such study in which the analysis of cell survival and proliferation on transplanted hAD was conducted in situ. The use of dorsal skin-fold chambers and in situ fluorescence microscopy provides an opportunity to monitor cell survival and proliferation in vivo over a period of 9 days. We are interested in the use of cell-seeded hAD within the context of multi-modal oncological treatment regimens and developed the experimental model described in this paper in order to track the fate of seeded cells in the early post-implantation period and to predict the effect of neoadjuvant radiation therapy on cell-seeded hAD.

After implantation there was an initial phase in which viable cell numbers declined. In all 4 treatment groups this persisted for at least 3 days.

Long-term survival of multiple cell layers in such transplants requires neovascularization. In the absence of a vascular network seeded cells will be primarily nourished by diffusive and possibly by convective mass transport. Cellular nutrient consumption rates, the number of cell layers requiring nutrition and the magnitude of the diffusive transport component will limit the length of the transport path that can be adequately supplied with nutrients in the absence of a vascular network. Since neovascularization of such implants progresses over several days to weeks, depending on the implantation environment and the size of the implant [38], it can be postulated that inadequate nutrient transport may be a factor involved in the cell depletion effect observed at day 3.

At day 6 there was a significant increase in cell numbers in non-irradiated subjects, but not in subjects that received radiation simulating a neoadjuvant therapy. The effect in irradiated subjects might be a result of radiation-induced damage of host endothelium leading to impairment of angeogenic function [39–42]. It may be the case that at this time point the onset of neoangeogensis in the non-irradiated groups is a factor contributing to increased numbers of viable fibroblasts.

The presence of pores in matrices for cell-seeding can have a positive effect on survival and proliferation of seeded cells. Previous studies describe this effect with acellular grafts with naturally occurring pores [43, 44] and synthetic scaffolds with pores produced with a laser [45]. One study describes a laser machining method for generating micropores in synthetic grafts that does not impair biomechanics [46].

This study demonstrates that the presence of hair follicle pores had a beneficial effect on cell proliferation at days 6 and 9 after implantation in both non-irradiated and pre-irradiated subjects. In all subjects that received implants containing hair follicle pores, a major component of the surviving and proliferating fibroblast population was located in the vicinity of the pores.

We hypothesize that nutrient transport from the wound bed to the implant surface may be enhanced in the pore channels. There may be an increased convective transport component here since convective currents will exist in the underlying host tissue and their propagation will be less restricted in the pore channels than within the matrix. It may also be the case that these pores provide natural channels for neovascularisation. The surface of the pores may have an extracellular matrix composition that is well suited to endothelial infiltration. However, further investigation is required in order to eluciduate the mechanisms involved.

### Limitation of the study

The size of an implantable biomaterial is limited by the area of the chamber observation window. In clinical practice much larger transplants will be used. The duration of the study is limited to 9 days post-implantation. There may be relevant effects beyond this time point.

## Conclusions

The present study demonstrates that fibroblasts seeded on a human dermis transplant can survive and proliferate in a dorsal skinfold chamber. Radiation treatment intended to mimic a multi-modal neoadjuvant therapeutic approach had a significant effect on the cell survival. Compared to the non-irradiated animals, the cell survival and proliferation onto the surface of the hAD after irradiation was significantly lower, particularly in non-porous matrices. The presence of hair follicle pores in the hAD resulted in significant increased cell proliferation in the vicinity of the pores in both non-irradiated and irradiated subjects. Transplant porosity may therefore be an important parameter for tissue engineered products intended for use in neoadjuvant settings.
